# Context-Dependent Modulation of Lipolytic Activity by *Ginkgo biloba* Extract: Evidence from Murine and Human Adipose Models

**DOI:** 10.3390/ph19101583

**Published:** 2026-10-07

**Authors:** Iomara Isidorio Cavalcante, Crislaine das Graças de Almeida, Fernanda Malanconi Thomaz, Jussara de Jesus Simão, Lucas Humberto Zimmermann Belaunde, Lila Missae Oyama, Eliane Beraldi Ribeiro, Maria Isabel Cardoso Alonso-Vale, Mônica Marques Telles

**Affiliations:** 1 Graduate Program in Nutrition, Discipline of Nutrition Physiology, Department of Physiology, Universidade Federal de São Paulo-UNIFESP, São Paulo 04023-062, Brazil; i.cavalcante@unifesp.br (I.I.C.); crislainedealmeida@gmail.com (C.d.G.d.A.); lmoyama@unifesp.br (L.M.O.); eliane.beraldi@gmail.com (E.B.R.); alonso.vale@unifesp.br (M.I.C.A.-V.); 2 Graduate Program in Chemical Biology, Institute of Environmental Sciences, Chemical and Pharmaceutical, Universidade Federal de São Paulo-UNIFESP, Diadema 09972-270, Brazil; fernanda.malanconi@gmail.com (F.M.T.); jussara.simao@unifesp.br (J.d.J.S.); lucas.belaunde@unifesp.br (L.H.Z.B.)

**Keywords:** *Ginkgo biloba* extract, lipolysis, adipocyte metabolism, white adipose tissue, hormone-sensitive lipase, perilipin-1

## Abstract

**Background/Objectives:** Dysregulated lipolysis contributes to adipose tissue dysfunction in obesity. *Ginkgo biloba* extract (GbE) influences adipose metabolism, but its effects across adipose models remain unclear. We evaluated GbE effects on lipolytic activity in murine and human models. **Methods:** Differentiated 3T3-L1 and hWA A41 adipocytes, primary adipocytes from one individual with obesity, and human visceral adipose tissue explants from individuals with overweight or obesity were exposed to GbE (1.00 mg/mL, 48 h). Lipolytic activity was assessed by glycerol release. *Plin1*, *Pnpla2*, and *Lipe* expression, ATGL and pHSL abundance, and MTT-based metabolic activity were evaluated. **Results:** GbE increased basal glycerol release nearly fivefold in 3T3-L1 adipocytes (*p* = 0.0045) and by 26.3% in hWA A41 cells (*p* = 0.0007). GbE reduced *Plin1* (*p* = 0.0182) and *Lipe* (*p* = 0.0027) expression, while *Pnpla2* remained unchanged. In hWA A41 cells, pHSL increased (*p* = 0.0459), while ATGL was unchanged. MTT-based metabolic activity increased at 1.00 mg/mL but decreased at higher concentrations. A descriptive 35% reduction in glycerol release was observed in primary adipocytes from one donor. GbE reduced glycerol release by approximately 45% in visceral adipose tissue explants from individuals with obesity, with no significant change in overweight. **Conclusions:** GbE produced distinct effects across adipose models, increasing lipolytic activity in adipocyte cell lines while reducing glycerol release in visceral adipose tissue explants from individuals with obesity, with a directionally similar reduction descriptively observed in primary adipocytes. These preliminary findings warrant further mechanistic and in vivo studies.

## 1. Introduction

Obesity has reached alarming proportions worldwide, representing a major and growing threat to global health [1]. This condition is characterized by excessive adiposity and is closely associated with the progressive expansion and remodeling of white adipose tissue (WAT). Under sustained energy surplus, WAT adapts to increased lipid storage demands through adipocyte hypertrophy and hyperplasia. However, when its capacity for healthy expansion is exceeded, adipose tissue undergoes structural and functional alterations that promote cellular stress, inflammation, and metabolic dysfunction, thereby contributing to obesity-related complications [1,2].

With increasing adiposity, alterations in adipose tissue function may disrupt lipid turnover, leading to a marked decline in the ratio of fatty acid turnover to lipolysis [2,3]. As dysfunctional adipose tissue becomes unable to accommodate further lipid storage, increased fluxes of free fatty acids into the circulation favor lipid deposition and the development of lipotoxicity. In addition, reduced neo-adipogenesis and dysfunctional lipid-overloaded adipocytes are characteristic of hypertrophic adipose tissue and are strongly linked to systemic insulin resistance [3].

These alterations in lipid handling are closely associated with dysregulation of the molecular machinery controlling lipolysis [4,5]. Perilipin-1 regulates lipase accessibility to lipid droplets, ATGL (*Pnpla2*) initiates triglyceride hydrolysis, and HSL (*Lipe*) acts predominantly on diacylglycerols, with its activity regulated in part by phosphorylation [4,5,6,7,8]. Together, these proteins are key regulators of adipocyte lipolytic activity [4,5].

Given the contribution of adipose tissue dysfunction to metabolic complications, reducing excess adiposity can counteract several of these abnormalities. Weight loss promotes a decrease in adipocyte size and helps restore metabolic flexibility by improving substrate utilization and oxidative metabolism, ultimately favoring metabolic homeostasis [9]. Nevertheless, lifestyle interventions alone generally produce relatively limited and difficult-to-maintain reductions in body weight, typically ranging from 2% to 9% after one year, while weight regain remains common despite continued treatment [10]. These challenges support the search for complementary therapeutic approaches capable of enhancing and sustaining metabolic benefits, including pharmacological, surgical, and phytotherapeutic strategies. Within the latter group, standardized *Ginkgo biloba* extract (GbE) has attracted attention because of its broad biological activities and has been the focus of investigation by our research group for more than a decade.

Our previous findings evidenced that GbE, a standardized preparation containing flavonoids and terpene lactones, can influence adipose tissue metabolism in experimental models. In diet-induced obese rats, treatment with GbE was associated with smaller adipocytes in both epididymal and retroperitoneal fat depots and reduced acetate accumulation, whereas ^3^H-oleate incorporation showed a non-significant tendency to decrease, suggesting suppression of de novo lipid synthesis. In addition, GbE modulated the abundance of proteins involved in adipocyte differentiation, carbon metabolic pathways, and mitochondrial activity [11,12]. Consistent with these observations, studies in 3T3-L1 cells demonstrated that GbE enhanced preadipocyte differentiation and upregulated adipogenic and cell proliferation markers at both the gene and protein levels [13]. However, whether GbE exerts comparable metabolic effects in human adipocytes and preserved human white adipose tissue (WAT) has not yet been fully established.

Given the limited understanding of how GbE affects adipocyte metabolism, we hypothesized that GbE modulates lipolytic activity and that the magnitude and/or direction of this response may differ among adipose experimental systems with distinct cellular and metabolic characteristics. To test this hypothesis, the present study evaluated the influence of standardized GbE on lipolytic activity across complementary experimental systems, including murine 3T3-L1 adipocytes, the human hWA A41 adipocyte cell line, primary human adipocytes obtained from one individual with obesity, and visceral WAT explants obtained from individuals with overweight and obesity. This approach was designed to characterize the response to GbE across adipose models with distinct biological characteristics, rather than to isolate the contribution of any single determinant.

## 2. Results

### 2.1. GbE Increased Glycerol Release and Modulated the Expression of Lipid Droplet–Associated Genes in 3T3-L1 Cells

Basal lipolytic activity in 3T3-L1 cells is shown in Figure 1A and was assessed by measuring glycerol release after treatment with GbE (1.00 mg/mL) for 48 h, following 7 days of adipocyte differentiation. Compared with control cells, GbE treatment resulted in an approximately fivefold increase in basal lipolysis (*p* = 0.0045).

RT-qPCR was used to assess the expression of three genes related to lipid metabolism. The selected genes are summarized in Table 1, and their expression profiles are shown in Figure 1B–D. GbE treatment significantly decreased *Plin1* (Perilipin 1) expression by 33% (*p* = 0.0182) and *Lipe* (Hormone-Sensitive Lipase) expression by 41% (*p* = 0.0027). In contrast, *Pnpla2* (Adipose Triglyceride Lipase) expression was not significantly affected by GbE (*p* = 0.1111).

### 2.2. GbE Treatment Concentration-Dependently Altered MTT-Based Metabolic Activity in hWA A41 Adipocytes

Figure 2 shows the effects of 48 h of GbE exposure on MTT-based metabolic activity in hWA A41 adipocytes. Compared with the control condition, treatment with 1.00 mg/mL GbE increased the MTT signal, whereas no significant changes were observed at 0.75 or 1.25 mg/mL. In contrast, higher concentrations of GbE (1.50 and 2.00 mg/mL) reduced MTT-based metabolic activity. Thus, the concentration selected for the subsequent experiments (1.00 mg/mL) did not reduce cellular MTT reducing activity. Because the MTT assay reflects cellular reducing capacity and may be influenced by both cell number and metabolic state, these findings should not be interpreted as a direct measurement of cell viability or mitochondrial function.

### 2.3. GbE Increased Glycerol Release in hWA A41 Cells Under Basal and Isoproterenol-Stimulated Conditions

Figure 3A shows glycerol release in hWA A41 cells under basal and isoproterenol-stimulated conditions after exposure to GbE (1.00 mg/mL) for 48 h. Two-way ANOVA revealed significant main effects of GbE treatment (F(1,8) = 15.56, *p* = 0.0043) and isoproterenol stimulation (F(1,8) = 28.26, *p* = 0.0007), whereas the GbE × isoproterenol interaction was not significant (F(1,8) = 3.559, *p* = 0.0959).

Figure 3B presents a broader assessment of basal glycerol release based on data obtained from several independent experiments. Following 12 days of differentiation, hWA A41 cells were exposed to GbE (1.00 mg/mL) for 48 h. Across this larger experimental dataset, GbE increased basal glycerol release by 26.3% compared with untreated cells (*p* = 0.0007). Thus, the approximately 45% increase shown in Figure 3A and the 26.3% increase shown in Figure 3B derive from distinct experimental datasets, with Figure 3B providing the more representative estimate of the basal response to GbE.

### 2.4. GbE Treatment Increased the Abundance of HSL Phosphorylated at Ser563 in hWA A41 Adipocytes

Western blot analysis was used to quantify ATGL and HSL phosphorylated at Ser563 (pHSL Ser563), two proteins associated with the lipolytic pathway, as shown in Figure 4A,B. ATGL protein abundance was not significantly altered by GbE treatment (*p* = 0.5673). In contrast, pHSL Ser563 abundance was 44.3% higher in the GbE-treated group than in the control group (*p* = 0.038). Because total HSL was not measured, these data do not permit the determination of the pHSL/total HSL ratio or direct assessment of HSL enzymatic activation.

### 2.5. GbE Treatment Reduced Basal Glycerol Release in Visceral WAT Explants from Participants with Obesity

De-identified individual demographic, anthropometric, and metabolic characteristics of the participants are provided in Supplementary Table S1.

Figure 5 summarizes the effects of GbE on basal glycerol release in human adipose tissue models. BMI, adipocyte volume, circulating leptin, MCP-1, and LPS concentrations are provided to further characterize the metabolic and inflammatory profiles of the participants.

In primary adipocytes isolated from the visceral adipose tissue of one individual with class I obesity, mean basal glycerol release was 35% lower in GbE-treated wells than in control wells (Figure 5A). The donor exhibited an elevated adipocyte volume that was higher than the mean value observed in the overweight group but lower than that observed in the obesity group (Figure 5B and Figure 5C, respectively). Circulating leptin and LPS concentrations were similar to those observed in the obesity group. Because all culture wells were derived from the same donor, they represent technical replicates rather than independent biological observations. Accordingly, these findings, including the comparisons with the overweight and obesity groups, are presented solely as descriptive observations, and no inferential statistical analysis was performed. The purpose of this descriptive analysis was to explore whether the direction of the response observed in WAT explants was also reflected in isolated primary visceral adipocytes.

In visceral WAT explants obtained from individuals with overweight, GbE did not significantly affect basal glycerol release (Figure 5B; n = 3 donors; *p* = 0.3801). In contrast, GbE reduced basal glycerol release by approximately 45% in WAT explants obtained from individuals with obesity (Figure 5C; n = 5 donors; *p* = 0.0263). For these analyses, replicate explant-derived wells were averaged within each donor and experimental condition, and the donor-level values were used for statistical comparisons. Compared with participants with overweight, those with obesity had higher BMI (+58%; *p* = 0.0015), adipocyte volume (+216.1%; *p* = 0.0011) and circulating concentrations of leptin (+196%; *p* < 0.0001) and MCP-1 (+123.6%; *p* = 0.0139).

## 3. Discussion

The present study showed that GbE produced distinct effects on basal glycerol release across adipose experimental models, increasing it in 3T3-L1 and hWA A41 adipocyte cell lines while reducing it in visceral WAT explants from individuals with obesity. A directionally similar reduction was descriptively observed in primary visceral adipocytes obtained from one individual with obesity. These findings extend previous observations from our group showing that GbE modulates adipose tissue metabolism in experimental models of metabolic dysfunction [11,12]. Because the models evaluated here differ in several biological and experimental characteristics, the divergent responses cannot be attributed to a single determinant but rather indicate that the effects of GbE vary across adipose experimental systems.

Consistent with the distinct metabolic profile of these participants, individuals with obesity from whom the WAT explants were obtained exhibited greater adipocyte volume and higher circulating concentrations of leptin and MCP-1 than individuals with overweight, along with a trend toward increased LPS concentrations. These biomarkers are commonly associated with adipose tissue dysfunction, chronic low-grade inflammation, and metabolic endotoxemia [14,15,16]. Such alterations may influence adipose tissue metabolism and the regulation of lipolysis [17,18]. However, given the limited number of participants and the absence of correlation or mechanistic analyses, the present findings do not establish a direct relationship between these circulating biomarkers and the GbE-induced changes in glycerol release.

Notably, GbE increased glycerol release in hWA A41 cells under both basal and isoproterenol-stimulated conditions. In 3T3-L1 adipocytes, increased basal glycerol release occurred despite the downregulation of *Plin1* and *Lipe* mRNA levels, suggesting that the response to GbE may involve regulatory mechanisms beyond changes in gene expression. In hWA A41 cells, the concentration used in the functional experiments (1.00 mg/mL) did not reduce MTT-based metabolic activity and was associated with an increased MTT signal, consistent with our previous observations [13].

The present findings show that GbE increased basal glycerol release in both adipocyte cell lines. At the molecular level, GbE reduced *Plin1* and *Lipe* mRNA levels in 3T3-L1 adipocytes, whereas *Pnpla2* expression remained unchanged. Perilipin-1 is an important regulator of lipid droplet accessibility to lipases; therefore, the reduction in *Plin1* expression may reflect altered regulation of the lipid droplet, although corresponding changes at the protein level were not assessed. In hWA A41 cells, GbE increased phosphorylated HSL levels without altering ATGL abundance. Although these findings were obtained in different cell models, they suggest that the effects of GbE on glycerol release may involve regulatory mechanisms beyond changes in gene transcription. However, because total HSL, PKA activity, and protein translocation were not evaluated, a specific signaling mechanism cannot be established. These observations are consistent with previous studies examining the effects of whole GbE and individual constituents on lipolysis-related pathways [11,19,20,21,22].

Previous findings from our group showed that GbE supplementation in rats with diet-induced obesity reduced adipocyte size and *Plin1* mRNA expression without altering basal or stimulated lipolysis or HSL/pHSL protein levels [11], indicating that changes in adipocyte morphology and lipolysis-related markers do not necessarily translate into altered lipolytic activity. Studies with isolated GbE constituents also support modulation of lipolysis-related pathways. Ginkgolide C increased glycerol release and altered ATGL and HSL expression in 3T3-L1 adipocytes [19], while GbE-derived biflavonoids enhanced basal lipolysis [20]. Bilobalide likewise modulated ATGL expression and HSL phosphorylation, with effects varying according to concentration [21]. Together, these findings further suggest that the effects of GbE and its constituents on adipocyte lipid metabolism are dependent on the experimental conditions.

The anatomical origin of the human adipose tissue evaluated here should also be considered, as WAT depots differ in their structural and metabolic characteristics [23,24,25,26]. Both the primary adipocytes and tissue explants were obtained from visceral WAT. The primary adipocyte experiment was included as a complementary approach to explore whether the direction of the response observed in intact WAT explants was also reflected at the isolated adipocyte level. In cells obtained from a donor with class I obesity, mean basal glycerol release was 35% lower following GbE treatment, which was directionally consistent with the inhibitory effect observed in visceral WAT explants from participants with obesity. The donor also exhibited a relatively high adipocyte volume, with a value higher than the mean observed in the overweight group but lower than that observed in the obesity group, whereas circulating leptin and LPS concentrations were similar to those found in the obesity group. Thus, although purely descriptive, these findings indicate qualitative correspondence between the responses observed in intact WAT explants and isolated primary visceral adipocytes.

Unlike isolated adipocytes, adipose tissue explants preserve tissue architecture and interactions among adipocytes and other resident cell populations involved in metabolic regulation [27]. In metabolically dysfunctional adipose tissue, impaired insulin-mediated suppression of lipolysis may coexist with increased basal lipolytic activity in hypertrophic adipocytes [17]. When adipose tissue storage capacity is exceeded, altered lipid handling may increase fatty acid flux and favor lipid overflow and ectopic lipid deposition in non-adipose tissues, thereby contributing to lipotoxicity, insulin resistance, and inflammation [3,18]. In this context, attenuation of excessive basal lipolysis by GbE could represent a potentially beneficial effect by limiting fatty acid overflow from adipose tissue and its downstream metabolic consequences. Nevertheless, because only glycerol release was measured ex vivo, without complementary assessment of free fatty acid release, re-esterification, or systemic metabolic outcomes, this potential benefit remains hypothetical and requires confirmation in mechanistic and in vivo studies. Although the characteristics of the primary adipocyte donor are compatible with the possibility that increasing BMI is accompanied by progressive adipocyte hypertrophy and a less favorable metabolic and inflammatory profile, the single-donor experiment and the descriptive comparisons across groups do not establish a BMI-dependent association or demonstrate that the response to GbE is determined by the donor’s metabolic status. Studies involving primary adipocytes from multiple donors across different BMI categories are required to investigate these relationships and determine the contribution of adipocytes and other tissue-resident cell populations to the observed response.

Several limitations should be considered. The GbE concentration used (1.00 mg/mL) was selected as an experimental in vitro/ex vivo exposure and should not be interpreted as representative of systemic concentrations achieved after oral administration in humans. In addition, lipolytic activity was not evaluated across multiple concentrations or exposure times. Although the standardized extract was chemically characterized, the contribution of individual constituents to the observed effects cannot be determined. Lipolytic activity was assessed by glycerol release alone, without complementary measurements of free fatty acid release, intracellular triglyceride content, or re-esterification. Mechanistic interpretation was also limited by the absence of total HSL measurements, which precluded calculation of the pHSL/HSL ratio, and by the lack of cAMP and PKA activity assessments. The primary adipocyte experiment was performed using cells from a single donor and should therefore be interpreted as a descriptive within-donor observation. Moreover, the limited number of participants in the explant experiments restricts population-level inference and the assessment of adiposity-group-specific responses. Accordingly, the divergent effects observed across models cannot be attributed to obesity, inflammation, or any single biological determinant. Further dose-ranging, mechanistic, pharmacokinetic, and in vivo studies are required to establish the biological and translational relevance of these findings.

GbE increased basal glycerol release in murine and human adipocyte cell lines under standardized in vitro conditions, whereas it reduced glycerol release in visceral adipose tissue explants from individuals with obesity, with a directionally similar decrease observed descriptively in isolated primary visceral adipocytes from one individual with obesity. These contrasting responses suggest that the effects of GbE vary according to the experimental and biological context of the adipose model. However, given the differences among the systems evaluated and the limitations of the human experiments, these findings should be considered preliminary. Further mechanistic, dose-ranging, pharmacokinetic, and in vivo studies are required to clarify the biological significance and translational relevance of GbE-induced modulation of adipose tissue lipolytic activity.

## 4. Materials and Methods

For all cell-line assays, an independent experiment was defined as a separately initiated culture and, when applicable, differentiation procedure performed on a distinct experimental occasion. Multiple wells analyzed within the same experiment and condition were considered technical replicates and were averaged to generate one experiment-level value per condition. Accordingly, n represents the number of independent experiments rather than the number of culture wells. The numbers of independent experiments and technical replicate wells used in each assay are specified in the corresponding figure legends.

### 4.1. Murine Cell Line

#### Differentiation of 3T3-L1 Preadipocytes

The differentiation protocol lasted seven days. Cells were initially cultured until full confluence, which was designated day −2 (D−2). Two days later, on day 0 (D0), the growth medium was replaced with Dulbecco’s Modified Eagle Medium/Ham’s F-12 (DMEM/Ham’s F-12) containing an adipogenic induction mixture composed of 0.5 mM 3-isobutyl-1-methylxanthine (IBMX), 0.1 μM dexamethasone, 2 nM T3, 30 μM indomethacin, 17 μM pantothenate, 33 μM biotin, 1 μM rosiglitazone, 1 μM transferrin (Sigma-Aldrich, St. Louis, MO, USA), 0.5 μM human insulin (Humulin, 100 IU/mL; Eli Lilly, Indianapolis, IN, USA), and 1% penicillin–streptomycin. Cells were maintained under these adipogenic conditions until day 7 (D7).

### 4.2. Human Cell Line hWA A41

#### Differentiation of Preadipocytes

The hWA A41 immortalized human adipocyte cell line was provided by the Joslin Diabetes Center (Boston, MA, USA) to the Federal University of São Paulo (UNIFESP, Brazil) under a material transfer agreement signed in April 2021. The agreement identifies Yu-Hua Tseng as the Joslin scientist and Lucia Maria Armelin Correa as the recipient scientist and permits research use in the recipient scientist’s laboratory under her direct supervision.

Human hWA A41 preadipocytes, which originate from subcutaneous adipose tissue of the neck, were immortalized by expressing human telomerase reverse transcriptase (hTERT) [28,29]. Before differentiation, the cells were routinely maintained at 37 °C in a humidified atmosphere containing 5% CO_2_. The culture medium consisted of high-glucose Dulbecco’s Modified Eagle Medium (DMEM; 4.5 g/L glucose) containing 10% fetal bovine serum (FBS) and 1% penicillin–streptomycin. Complete confluence was defined as day 0 (D0), when adipogenic differentiation was induced. For the subsequent 12-day differentiation period, high-glucose DMEM was supplemented with 33 μM biotin, 17 μM pantothenate, 30 μM indomethacin, 2 nM T3, 0.5 μM insulin, 0.1 μM dexamethasone, 0.5 mM 3-isobutyl-1-methylxanthine (IBMX), 10% FBS, and 1% penicillin–streptomycin. Fresh differentiation medium was provided at three-day intervals until day 12 (D12).

The hWA A41 differentiation medium described above was also used during the 48 h experimental treatment of isolated primary human adipocytes and visceral WAT explants, as detailed in Section 4.3.3 and Section 4.3.4, respectively.

### 4.3. Primary Adipocytes and Tissue Explant

#### 4.3.1. Participant Selection and Visceral Adipose Tissue Collection

The collection of human adipose tissue and blood was approved by the Human Research Ethics Committee of the Universidade Federal de São Paulo and the Ethics Committee of Hospital Municipal de Paulínia (HMP-SP). During outpatient evaluation, the surgical team screened potential participants, explained the study procedures, and invited eligible individuals to participate. Written informed consent was obtained from all participants before enrollment. For the visceral WAT explant experiments, eligibility criteria included male sex, age between 28 and 50 years, body mass index (BMI) > 25 kg/m^2^, and scheduled nephrectomy, cystectomy, or prostatectomy. Participants were excluded if they smoked, had cancer or a genetic disorder, had experienced recent weight loss, or were using drugs, hormones, or other medications known to affect white adipose tissue morphology or function. Eight participants were included and classified according to BMI as having overweight (*n* = 3) or obesity (*n* = 5). An additional visceral WAT sample from a 69-year-old patient with obesity was used exclusively for the single-donor primary adipocyte experiment described in Section 4.3.3 and was not included in the explant group comparisons. During surgery, approximately 15 g of visceral adipose tissue was collected and immediately placed in phosphate-buffered saline supplemented with penicillin–streptomycin. Samples were processed within 3 h of collection [30].

#### 4.3.2. Serum Dosages

Blood samples were centrifuged at 3000× *g* for 20 min at 4 °C, and the resulting serum was stored at −80 °C until analysis. Circulating lipopolysaccharide (LPS) levels were measured using the Pierce™ Chromogenic Endotoxin Quant Kit (Thermo Scientific, Waltham, MA, USA; catalog no. A39552S). Serum monocyte chemoattractant protein-1 (MCP-1) and leptin concentrations were quantified using specific enzyme-linked immunosorbent assay (ELISA) kits (Quantikine; R&D Systems, Minneapolis, MN, USA).

#### 4.3.3. Isolation of Primary Adipocytes from Human Visceral Adipose Tissue

Primary adipocytes were isolated from visceral WAT obtained from a single 69-year-old participant with class I obesity (PA1; Supplementary Table S1). This donor was included exclusively in the descriptive primary-adipocyte experiment and was not part of the explant group comparisons. The freshly collected tissue was processed within 3 h of surgery, and adipocytes were isolated according to the previously described protocol [30]. Following isolation, cells were distributed into 6–7 culture wells per experimental condition and maintained for 48 h in the hWA A41 differentiation medium described in Section Differentiation of Preadipocytes. Cells were maintained either in control medium without GbE or in the same medium containing 1.00 mg/mL GbE.

The donor constituted the biological unit (*n* = 1), whereas the individual culture wells represented technical replicates. Because all wells originated from the same donor, the experiment was treated as a descriptive within-donor analysis, and no population-level inferential statistical test was performed.

#### 4.3.4. Culture of Human Visceral Adipose Tissue Explants

For the adipose tissue explant experiments, freshly collected visceral WAT was cut into fragments weighing approximately 40 mg each. Three fragments were placed in each culture well, corresponding to approximately 120 mg of tissue per well. For each participant, replicate wells were allocated to the control and GbE-treated conditions and maintained for 48 h in the hWA A41 differentiation medium described in Section Differentiation of Preadipocytes, either without or with 1.00 mg/mL GbE. Wells from the same participant and experimental condition were considered technical replicates and were averaged to generate one donor-level value per condition. Thus, each participant represented an independent biological replicate. Glycerol release was normalized to tissue mass and incubation time and expressed as nmol·mg^−1^·h^−1^.

### 4.4. Treatment with Standardized Ginkgo biloba Extract (GbE)

The *Ginkgo biloba* extract (GbE) used in this study was supplied by Huacheng Biotech Inc. (Changsha, China). According to the manufacturer’s certificate of analysis, the extract comprised 6.62% terpenoids and 25.21% flavonoids. Within the terpenoid fraction, bilobalide accounted for 2.73%, whereas ginkgolides C, B, and A collectively represented 3.09%. The chemical composition of this extract was previously characterized by our re-search group using high-performance liquid chromatography coupled with mass spectrometry (HPLC/MS), which identified rutin, isorhamnetin, quercetin, and kaempferol [31].

For the 48 h experimental treatment, each model was maintained in the culture or maintenance medium specified in its respective subsection. GbE was added to this medium at a final concentration of 1.00 mg/mL, whereas the matched control samples were maintained in the identical medium without GbE. Samples were incubated for 48 h at 37 °C in a humidified atmosphere containing 5% CO_2_. The GbE concentration was selected based on a preliminary pilot study.

### 4.5. Lipolysis Assay

Following the 48 h model-specific treatment described above, cells and adipose tissue explants underwent a 4 h nutrient-deprivation period in insulin-free, low-glucose DMEM. Samples were subsequently washed with phosphate-buffered saline (PBS) and transferred to Glucose Transporter Buffer containing 20 mM ascorbic acid and 0.9 g/L glucose. After incubation on ice for 30 min, a 20 μL aliquot of medium was collected from each well.

Glycerol released into the medium was quantified using the Free Glycerol Determination Kit (Sigma-Aldrich, St. Louis, MO, USA) according to the manufacturer’s instructions. The assay reaction mixtures were incubated at 37 °C for 30 min, and absorbance was measured at 540 nm using a spectrophotometer. Glycerol release was expressed as nmol per 10^6^ cells for the adipocyte models and as nmol of glycerol per mg of adipose tissue per hour (nmol·mg^−1^·h^−1^) for the explant experiments. For the explant assays, each culture well contained three tissue fragments weighing approximately 40 mg each, corresponding to a total tissue mass of approximately 120 mg.

### 4.6. Gene Expression Analysis by RT-qPCR

RNA extraction from 3T3-L1 cells was carried out with TRIzol^®^ reagent (Invitrogen Life Technologies, Waltham, MA, USA), using 1 mL for each 5–10 × 10^6^ cells. The extracted RNA was kept at −80 °C until the subsequent processing steps. Samples were further purified with a single-step, column-based purification kit supplied by Qiagen GmbH (Hilden, Germany). RNA yield and purity were determined spectrophotometrically using a NanoDrop™ instrument (Thermo Scientific, Waltham, MA, USA), with purity assessed from the A260/A280 and A260/A230 absorbance ratios.

Total RNA was reverse-transcribed into complementary DNA (cDNA) using the SuperScript III™ Reverse Transcriptase Kit (Invitrogen Life Technologies, Carlsbad, CA, USA). Gene expression was subsequently analyzed by quantitative real-time PCR (qPCR) with SYBR Green™ chemistry on a Rotor-Gene system (Qiagen GmbH, Hilden, Germany), according to a protocol reported previously [32]. Table 1 presents the primer sequences and corresponding annealing temperatures. Relative transcript levels were calculated by the comparative cycle threshold method (2^−ΔΔCt^), using 36b4 as the reference gene and the control group as the calibrator. The results are reported as target gene expression relative to 36b4.

### 4.7. MTT-Based Assessment of Metabolic Activity in hWA A41 Adipocytes

MTT-based metabolic activity was assessed in hWA A41 adipocytes as previously described [33]. Cells were seeded in 96-well plates at a density of 5 × 10^4^ cells/mL and, after 24 h, exposed to the GbE concentrations indicated in Figure 2 for 48 h at 37 °C and 5% CO_2_. The culture medium was then replaced with 90 µL of DMEM and 10 µL of MTT solution (5 mg/mL), followed by incubation at 37 °C for 3 h. The resulting formazan was dissolved in 100 µL of dimethyl sulfoxide (DMSO), and absorbance was measured at 570 nm. MTT-based metabolic activity was calculated relative to the untreated control, which was defined as 100% [34].

### 4.8. Protein Expression Analysis by Western Blotting

Differentiated hWA A41 cells cultured in 6-well plates (Corning Life Sciences, Tewksbury, MA, USA) were washed twice with PBS and lysed directly in the wells using radioimmunoprecipitation assay (RIPA) buffer containing a protease inhibitor cocktail (Roche Mini, Basel, Switzerland) prepared according to the manufacturer’s instructions. Cells were scraped from the wells, homogenized, transferred to 1.5 mL microcentrifuge tubes, and kept on ice. The soluble protein fraction was then aliquoted and stored at −80 °C until analysis.

Total protein content was determined using the bicinchoninic acid (BCA) assay. For Western blotting, 20 µg of protein per lane were denatured at 100 °C for 5 min and resolved on 12% or 15% polyacrylamide gels, as appropriate, for the detection of adipose triglyceride lipase (ATGL) and phosphorylated hormone-sensitive lipase (pHSL). The separated proteins were transferred to nitrocellulose membranes, and transfer efficiency was confirmed by Ponceau S staining.

Before immunodetection, membranes were blocked for 2 h with 5% bovine serum albumin (BSA) prepared in Tris-buffered saline containing Tween-20 (TBS-T). Membranes were then incubated overnight at 4 °C with rabbit primary antibodies against phospho-HSL (Ser563; #4139; 1:1000. Cell Signaling Technology, Danvers, MA, USA) or ATGL (#2138; 1:1000. Cell Signaling Technology), diluted in the blocking solution. After three washes with TBS-T for 10 min each, membranes were incubated for 1 h with a horseradish peroxidase-conjugated anti-rabbit secondary antibody (Sigma-Aldrich, A0545; 1:10,000). Membranes were subsequently washed three times with TBS-T for 10 min each. Ponceau S staining was used for the normalization of protein abundance. After the final washing step, the membranes were exposed to a chemiluminescent substrate, and the resulting signals were immediately captured using an imaging system. ATGL and pHSL band intensities were determined by densitometric analysis with ImageJ 1.54d software. For each sample, the target protein signal was normalized to the total protein staining obtained with Ponceau S in the corresponding lane. The normalized value of the untreated basal group was defined as 100%, and the results were expressed as a percentage of the basal condition.

### 4.9. Statistical Analysis

Data from independent biological replicates are presented as mean ± SEM. For cell-line experiments, independently performed culture and differentiation experiments constituted biological replicates, whereas technical wells were averaged within each experiment and condition. Normality was assessed using the Shapiro–Wilk test; for paired analyses, this assumption was evaluated using the differences between paired values. Homogeneity of variance was assessed using Levene’s test for unpaired comparisons. Serum parameters were analyzed using unpaired, two-tailed Student’s *t*-tests. Paired, two-tailed Student’s *t*-tests were used to analyze basal glycerol release in 3T3-L1 and hWA A41 adipocytes, ATGL abundance (Figure 4A), HSL phosphorylation (Figure 4B), and *Pnpla2* and *Lipe* expression in 3T3-L1 adipocytes. *Plin1* expression was analyzed using a paired, one-tailed Student’s *t*-test. For adipose tissue explants (Figure 5B,C), replicate-well measurements were averaged within each participant and condition, yielding one donor-level value per condition. Control and GbE-treated values from the same donor were compared using paired, two-tailed Student’s *t*-tests.

The single-donor primary adipocyte experiment (Figure 5A) was analyzed descriptively without population-level inference. Isoproterenol-stimulated glycerol release was analyzed using two-way ANOVA, with GbE treatment and isoproterenol stimulation as factors and the GbE × isoproterenol interaction included in the model, followed by Tukey’s multiple-comparisons test. MTT-based metabolic activity was analyzed using one-way ANOVA followed by Dunnett’s multiple-comparisons test. Gene expression data were additionally processed using Real-Time Ready Profiler PCR Array software v. 3.5 (SABiosciences, Qiagen GmbH, Hilden, Germany), according to the manufacturer’s instructions. Statistical analyses were performed using GraphPad Prism v.10 (GraphPad Software, San Diego, CA, USA), with statistical significance set at *p* < 0.05.

### 4.10. Graphical Abstract Preparation

The graphical abstract was generated using ChatGPT-5.6 Sol (OpenAI) based on an original prototype developed by the authors. The resulting image was subsequently reviewed and edited by the authors, who verified the accuracy of all visual elements and scientific information, and took full responsibility for the final content.

## Figures and Tables

**Figure 1:**
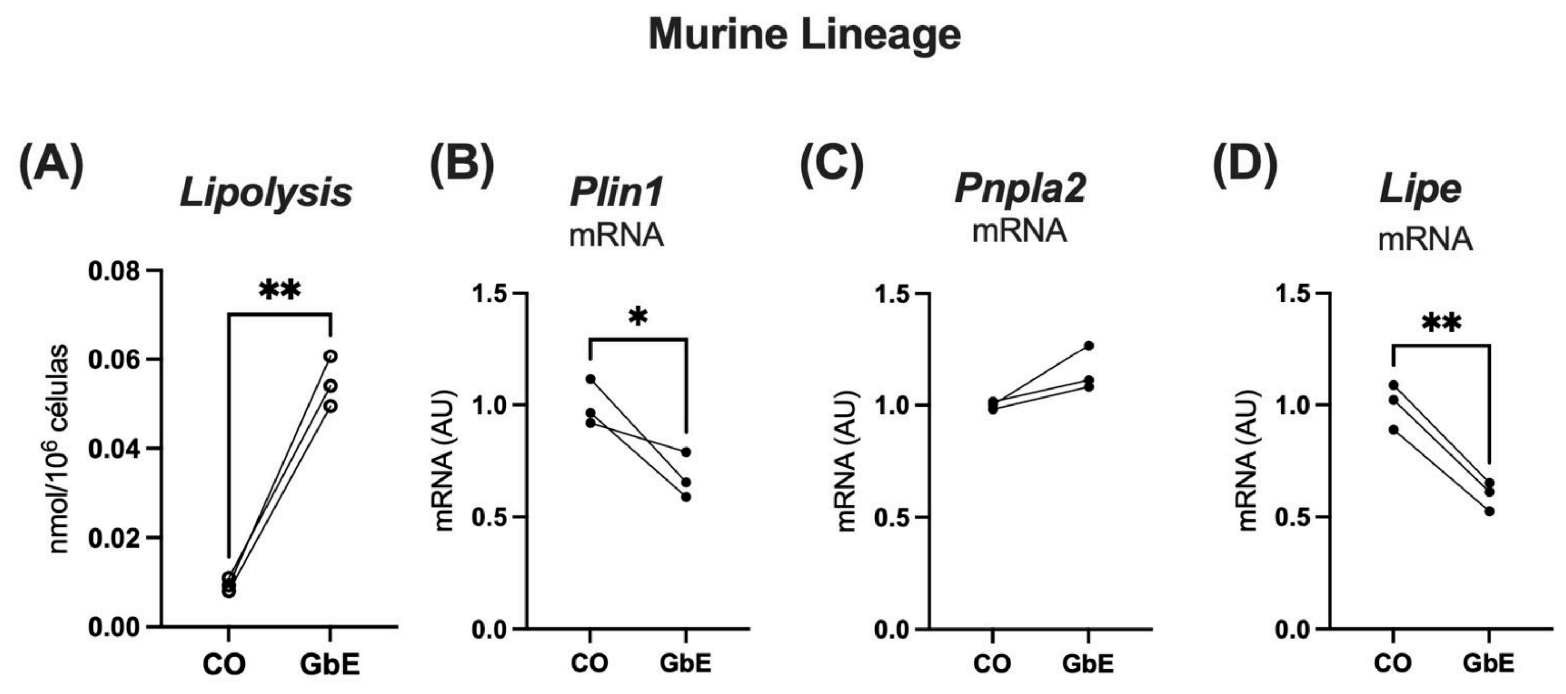
(**A**) Free glycerol release (nmol/10^6^ cells) was measured as an index of basal lipolysis in 3T3-L1 adipocytes differentiated for 7 days and subsequently incubated with or without GbE (1.00 mg/mL) for 48 h. Data were obtained from three independent experiments, each performed with 2–3 technical replicates (*n* = 3 independent experiments). (**B**–**D**) Relative mRNA levels of lipolysis-related genes were determined by RT-qPCR under the same experimental conditions (*n* = 3): (**B**) *Plin1* (Perilipin 1); (**C**) *Pnpla2* (Patatin-Like Phospholipase Domain Containing 2), also known as Adipose Triglyceride Lipase (ATGL); and (**D**) *Lipe* (Hormone-Sensitive Lipase). Data are presented as mean ± standard error of the mean (SEM). Differences between control and GbE-treated groups were analyzed using a paired one-tailed Student’s *t*-test for *Plin1* (panel B), and paired two-tailed Student’s *t*-tests for *Pnpla2* and *Lipe* (panels C and D, respectively). * *p* < 0.05 and ** *p* < 0.01 versus the control condition.

**Figure 2:**
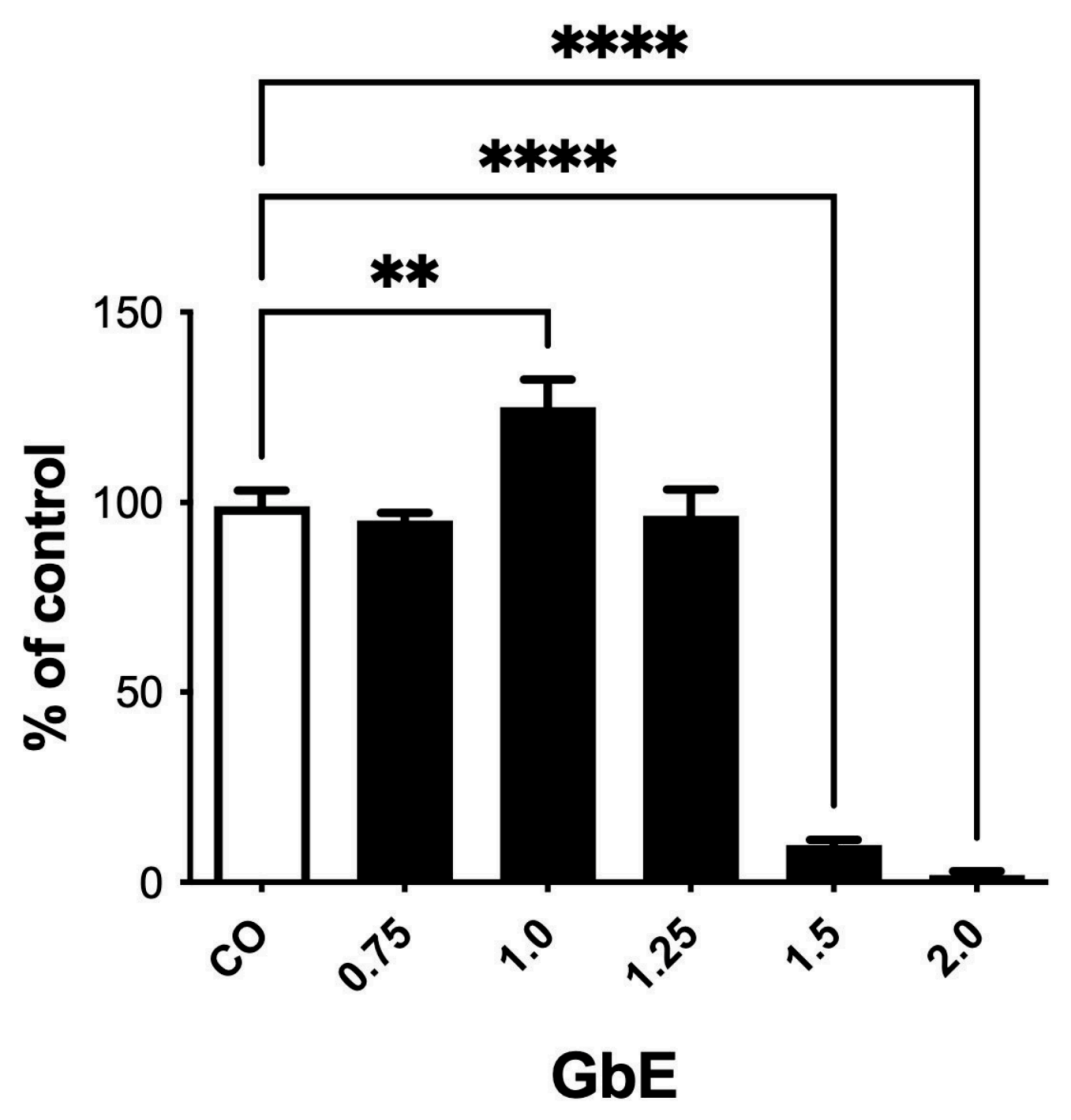
Effect of GbE on MTT-based metabolic activity in hWA A41 adipocytes. Cells were exposed to different concentrations of GbE or maintained under control conditions for 48 h. MTT reduction was expressed relative to the untreated control, which was set at 100%. Data are presented as mean ± standard error of the mean (SEM) (*n* = 4 independent experiments). Statistical differences among groups were assessed using one-way ANOVA followed by Dunnett’s multiple-comparisons test. ** *p* < 0.01 and **** *p* < 0.0001, versus the control condition.

**Figure 3:**
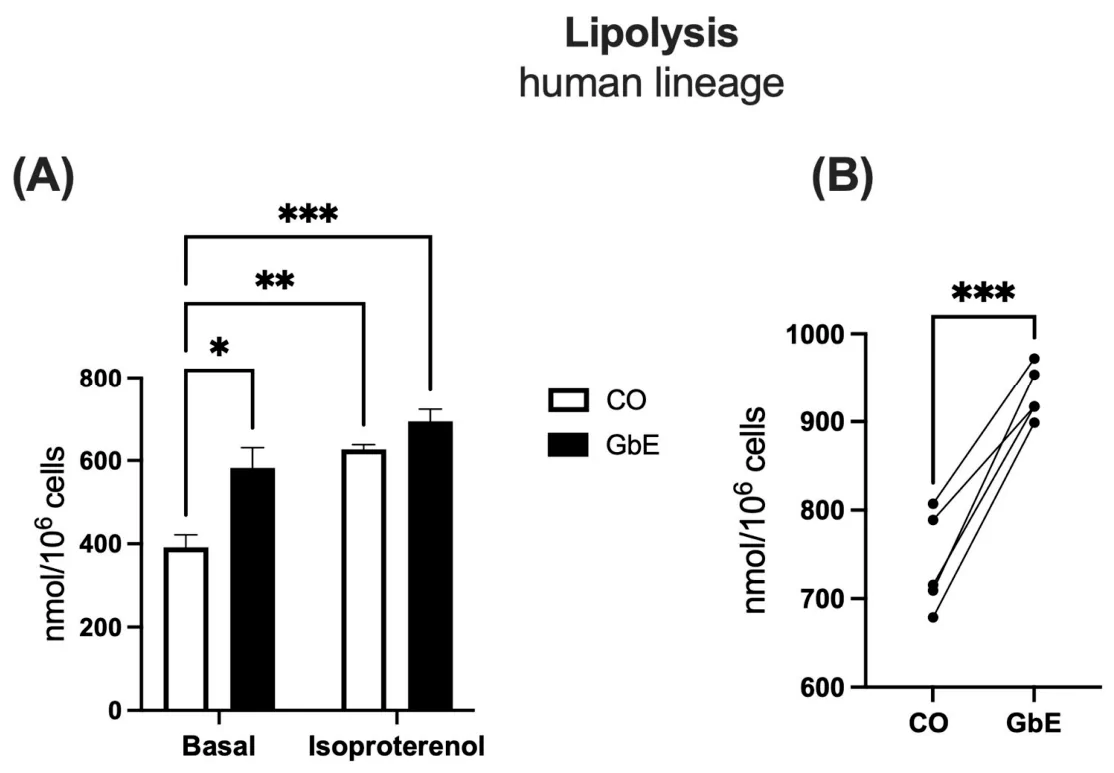
(**A**) Glycerol release was evaluated under basal and isoproterenol-stimulated conditions in hWA A41 adipocytes differentiated for 12 days and subsequently treated with GbE (1.00 mg/mL) or maintained under control conditions for 48 h. (**B**) Basal lipolytic activity was assessed under the same experimental conditions. Data were obtained from five independent experiments, each performed with 3–4 technical replicates (*n* = 5 independent experiments). In both panels, lipolysis was determined by quantification of free glycerol release and expressed as nmol/10^6^ cells. Data are presented as mean ± standard error of the mean (SEM). Statistical comparisons were performed using two-way ANOVA followed by Tukey’s post hoc test for panel (**A**) and paired two-tailed Student’s *t*-test for panel (**B**). Connecting lines indicate statistically significant differences between the indicated groups. * *p* < 0.05 vs. control; ** *p* < 0.01 vs. control; *** *p* < 0.001 vs. control vs. control.

**Figure 4:**
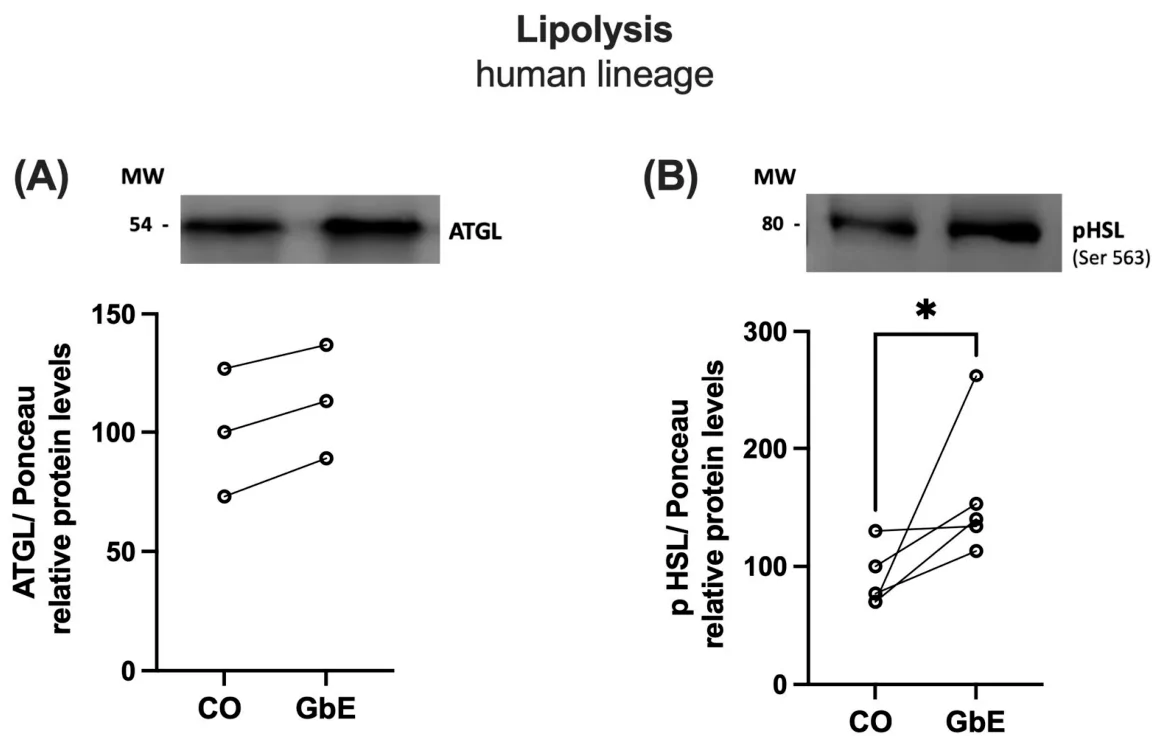
(**A**) Adipose triglyceride lipase (ATGL) protein abundance was determined in human hWA A41 adipocytes following a 12-day differentiation period and subsequent exposure to GbE (1.00 mg/mL) or control conditions for 48 h (*n* = 3 independent experiments). (**B**) The abundance of HSL phosphorylated at Ser563 (pHSL Ser563) was assessed under the same experimental conditions (*n* = 4–5 independent experiments). ATGL and pHSL Ser563 abundance were normalized to total protein based on Ponceau S staining and expressed relative to the respective control group. Data are presented as mean ± standard error of the mean (SEM). ATGL abundance (panel (**A**)) and pHSL Ser563 (panel (**B**)) were analyzed using a paired two-tailed Student’s *t*-test. HSL, hormone-sensitive lipase. * *p* < 0.05 versus the control condition.

**Figure 5:**
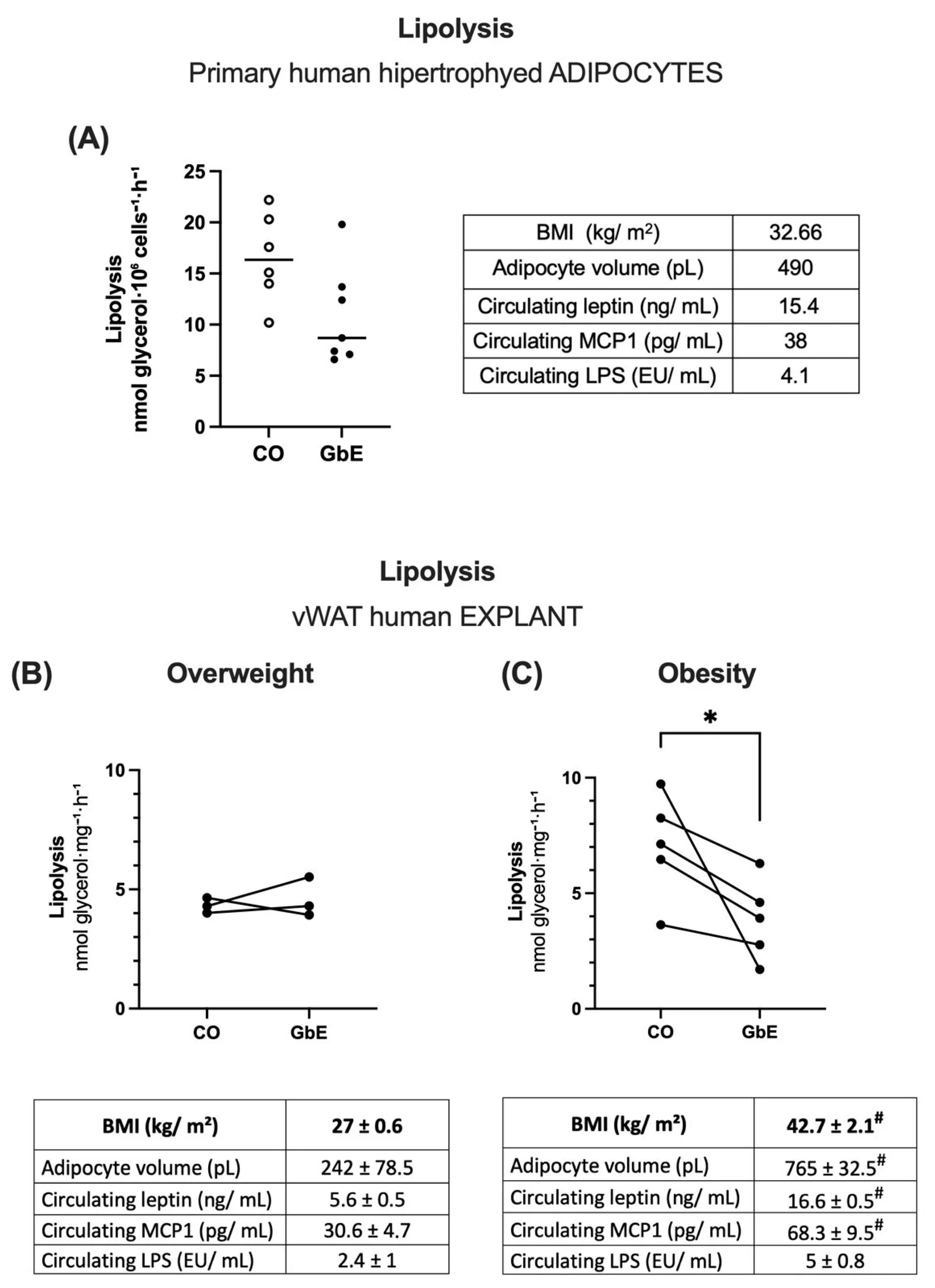
Effects of *Ginkgo biloba* extract (GbE) on basal glycerol release in human adipose tissue models. (**A**) Basal glycerol release was evaluated in primary adipocytes isolated from visceral white adipose tissue obtained from an individual with obesity. Cells were treated with GbE (1.00 mg/mL) or maintained under control conditions for 48 h, and lipolytic activity was determined by measuring free glycerol release (nmol/10^6^ cells). The individual culture wells represent technical replicates derived from a single donor (*n* = 1 donor; 6–7 technical replicates per condition). This experiment is presented descriptively, and no inferential statistical analysis was performed. (**B**) Basal glycerol release was evaluated in visceral white adipose tissue explants obtained from individuals with overweight (*n* = 3 independent biological replicates [donors], with four explant-derived culture wells analyzed per donor). (**C**) Basal glycerol release was evaluated in visceral white adipose tissue explants obtained from individuals with obesity (*n* = 5 independent biological replicates [donors], with 2–4 explant-derived culture wells analyzed per donor). For panels B and C, technical-well measurements were averaged within each donor and condition, and statistical comparisons were performed using donor-level values. Data are presented as mean ± standard error of the mean (SEM) across donors. Control and GbE-treated samples from the same donor were compared using a paired two-tailed Student’s *t*-test. * *p* < 0.05 versus the corresponding control condition. BMI, adipocyte volume, and circulating concentrations of leptin, MCP-1, and LPS are shown below the corresponding panels and are presented as mean ± SEM. Differences between participants with overweight and those with obesity were analyzed using an unpaired two-tailed Student’s *t*-test. # *p* < 0.05 versus the overweight group. vWAT, visceral White Adipose Tissue; MCP-1, monocyte chemoattractant protein-1; LPS, lipopolysaccharide; EU, endotoxin units.

**Table 1:** Sense and antisense sequences of primers evaluated in real-time RT-PCR.

Gene	5′ Primer (5′-3′) -Sense	3′ Primer (5′-3′) -Antisense	Annealing
*Plin1*	AGTGTGGGGTCCTTGGGCGT	TGGCAGCTGTGAACTGGGTGG	60 °C
*Pnpla2*	GGTCCTCTGCATCCCTCCTT	CTGTCCTGAGGGAGATGTC	60 °C
*Lipe*	CCTGCTGACCATCAACCGAC	CCTCGATCTCCGTGATATTCCA	60 °C

*Plin1*-Perilipin1; *Pnpla2* (ATGL) Patatin As Phospholipase Domain Containing 2; *Lipe*-Hormone-sensitive Lipase (HSL).

## Data Availability

The original contributions presented in this study are included in the article/Supplementary Material. Further inquiries can be directed to the corresponding author.

## Supplementary Materials

The following supporting information can be downloaded at https://www.mdpi.com/article/10.3390/ph19101583/s1: Table S1. Individual anthropometric and metabolic characteristics of male participants who provided visceral white adipose tissue for the explant and primary adipocyte experiments.

## References

[1] Hildebrand S., Pfeifer A. (2025). The obesity pandemic and its impact on non-communicable disease burden. Pflüg. Arch.-Eur. J. Physiol..

[2] Palacios-Marin I., Serra D., Jimenez-Chillarón J., Herrero L., Todorčević M. (2023). Adipose Tissue Dynamics: Cellular and Lipid Turnover in Health and Disease. Nutrients.

[3] Engin A., Engin A.B., Engin A. (2024). Lipid Storage, Lipolysis, and Lipotoxicity in Obesity. Obesity and Lipotoxicity.

[4] Bolsoni-Lopes A., Alonso-Vale M.I. (2015). Lipolysis and lipases in white adipose tissue—An update. Arch. Endocrinol. Metab..

[5] Frühbeck G., Méndez-Giménez L., Fernández-Formoso J.A., Fernández S., Rodríguez A. (2014). Regulation of adipocyte lipolysis. Nutr. Res. Rev..

[6] Holm C. (2003). Molecular mechanisms regulating hormone-sensitive lipase and lipolysis. Biochem. Soc. Trans..

[7] Tansey J.T., Sztalryd C., Hlavin E.M., Kimmel A.R., Londos C. (2004). The central role of perilipin a in lipid metabolism and adipocyte lipolysis. IUBMB Life.

[8] Sztalryd C., Xu G., Dorward H., Tansey J.T., Contreras J.A., Kimmel A.R., Londos C. (2003). Perilipin A is essential for the translocation of hormone-sensitive lipase during lipolytic activation. J. Cell Biol..

[9] Miranda A.M.A., McAllan L., Mazzei G., Andrew I., Davies I., Ertugrul M., Kenkre J., Kudo H., Carrelha J., Patel B. (2025). Selective remodelling of the adipose niche in obesity and weight loss. Nature.

[10] Yanovski S.Z., Yanovski J.A. (2024). Approach to Obesity Treatment in Primary Care: A Review. JAMA Intern. Med..

[11] Hirata B.K.S., Cruz M.M., de Sá R.D.C.C., Farias T.S.M., Machado M.M.F., Bueno A.A., Alonso-Vale M.I.C., Telles M.M. (2019). Potential anti-obesogenic effects of Ginkgo biloba observed in epididymal white adipose tissue of obese rats. Front. Endocrinol..

[12] Hirata B.K.S., Pedroso A.P., Machado M.M.F., Neto N.I.P., Perestrelo B.O., de Sá R.D.C.C., Alonso-Vale M.I.C., Nogueira F.N., Oyama L.M., Ribeiro E.B. (2019). Ginkgo biloba extract modulates the retroperitoneal fat depot proteome and reduces oxidative stress in diet-induced obese rats. Front. Pharmacol..

[13] Thomaz F.M., de Jesus Simão J., da Silva V.S., Machado M.M.F., Oyama L.M., Ribeiro E.B., Alonso Vale M.I.C., Telles M.M. (2022). Ginkgo biloba extract stimulates adipogenesis in 3T3-L1 preadipocytes. Pharmaceuticals.

[14] Ren Y., Zhao H., Yin C., Lan X., Wu L., Du X., Griffiths H.R., Gao D. (2022). Adipokines, hepatokines and myokines: Focus on their role and molecular mechanisms in adipose tissue inflammation. Front. Endocrinol..

[15] Izquierdo A.G., Crujeiras A.B., Casanueva F.F., Carreira M.C. (2019). Leptin, obesity, and leptin resistance: Where are we 25 years later?. Nutrients.

[16] Saad M.J., Santos A., Prada P.O. (2016). Linking gut microbiota and inflammation to obesity and insulin resistance. Physiology.

[17] Wueest S., Rapold R.A., Rytka J.M., Schoenle E.J., Konrad D. (2009). Basal lipolysis, not the degree of insulin resistance, differentiates large from small isolated adipocytes in high-fat-fed mice. Diabetologia.

[18] Muir L.A., Neeley C.K., Meyer K.A., Baker N.A., Brosius A.M., Washabaugh A.R., Varban O.A., Finks J.F., Zamarron B.F., Flesher C.G. (2016). Adipose tissue fibrosis, hypertrophy, and hyperplasia: Correlations with diabetes in human obesity. Obesity.

[19] Liou C.J., Lai X.Y., Chen Y.L., Wang C.L., Wei C.H., Huang W.C. (2015). Ginkgolide C suppresses adipogenesis in 3T3-L1 adipocytes via the AMPK signaling pathway. Evid. Based Complement. Altern. Med..

[20] Dell’Agli M., Bosisio E. (2002). Biflavones of Ginkgo biloba stimulates lipolysis in 3T3-L1 adipocytes. Planta Med..

[21] Bu S., Yuan C.Y., Xue Q., Chen Y., Cao F. (2019). Bilobalide suppresses adipogenesis in 3T3-L1 adipocytes via the AMPK signaling pathway. Molecules.

[22] Aziz T.A., Hussain S.A., Mahwi T.O., Ahmed Z.A., Rahman H.S., Rasedee A. (2018). The efficacy and safety of Ginkgo biloba extract as an adjuvant in type 2 diabetes mellitus patients ineffectively managed with metformin: A double-blind, randomized, placebo-controlled trial. Drug Des. Dev. Ther..

[23] Zwick R.K., Guerrero-Juarez C.F., Horsley V., Plikus M.V. (2018). Anatomical, physiological, and functional diversity of adipose tissue. Cell Metab..

[24] Wajchenberg B.L. (2000). Subcutaneous and visceral adipose tissue: Their relation to the metabolic syndrome. Endocr. Rev..

[25] Shuster A., Patlas M., Pinthus J.H., Mourtzakis M. (2012). The clinical importance of visceral adiposity: A critical review of methods for visceral adipose tissue analysis. Br. J. Radiol..

[26] Kwok K.H.M., Lam K.S.L., Xu A. (2016). Heterogeneity of white adipose tissue: Molecular basis and clinical implications. Exp. Mol. Med..

[27] Ràfols E.M. (2014). Adipose tissue: Cell heterogeneity and functional diversity. Endocrinol. Nutr..

[28] Xue R., Lynes M.D., Dreyfuss J.M., Shamsi F., Schulz T.J., Zhang H., Huang T.L., Townsend K.L., Li Y., Takahashi H. (2015). Clonal analyses and gene profiling identify genetic biomarkers of the thermogenic potential of human brown and white preadipocytes. Nat. Med..

[29] Leiria L.O., Wang C.-H., Lynes M.D., Yang K., Shamsi F., Sato M., Sugimoto S., Chen E.Y., Bussberg V., Narain N.R. (2019). 12-Lipoxygenase regulates cold adaptation and glucose metabolism by producing the omega-3 lipid 12-HEPE from brown fat. Cell Metab..

[30] Bellei B., Migliano E., Tedesco M., Caputo S., Picardo M. (2017). Maximizing non-enzymatic methods for harvesting adipose-derived stem cells from lipoaspirate: Technical considerations and clinical implications for regenerative surgery. Sci. Rep..

[31] Machado M.M.F., Banin R.M., Thomaz F.M., de Andrade I.S., Boldarine V.T., de Souza Figueiredo J., Hirata B.K.S., Oyama L.M., Lago J.H.G., Ribeiro E.B. (2021). Ginkgo biloba extract (GbE) restores serotonin and leptin receptor levels and plays an antioxidative role in the hippocampus of ovariectomized rats. Mol. Neurobiol..

[32] Tung Y.C., Hsieh P.H., Pan M.H., Ho C.T. (2017). Cellular models for the evaluation of the antiobesity effect of selected phytochemicals from food and herbs. J. Food Drug Anal..

[33] Mosmann T. (1983). Rapid colorimetric assay for cellular growth and survival: Application to proliferation and cytotoxicity assays. J. Immunol. Methods.

[34] Kwon S.S., Kim S.Y., Kong B.J., Kim K.J., Noh G.Y., Im N.R., Lim J.W., Ha J.H., Kim J., Park S.N. (2015). Cell-penetrating peptide conjugated liposomes as transdermal delivery system of Polygonum aviculare L. extract. Int. J. Pharm..

