# Supplementary material for: Context-Dependent Modulation of Lipolytic Activity by Ginkgo biloba Extract: Evidence from Murine and Human Adipose Models
Source: Pharmaceuticals (Basel). 2026 Oct 7;19(10):1583. doi: 10.3390/ph19101583 (PMC13708460; doi:10.3390/ph19101583)
Supplement: Supplementary file 1 [file pharmaceuticals-19-01583-s001.zip › pharmaceuticals-4519908-supplementary.pdf]

Supplementary Table S1. Individual anthropometric and metabolic characteristics of male participants who provided visceral white adipose tissue for the explant and primary adipocyte experiments.

**Participant**	**Group**	**Age (Years)**	**BMI (kg/m^2)**	**Leptin (ng/mL)**	**MCP-1 (pg/mL)**	**LPS (ng/mL)**	**Adipocyte volume (pL)**
PA1	Obesity	69	32.6	15.4	38.3	4.11	492.0
OW1	Overweight	28	28.0	6.6	40.0	0.43	392.3
OW2	Overweight	50	27.0	5.1	25.0	3.00	127.7
OW3	Overweight	30	25.9	5.0	26.7	3.66	206.0
OB1	Obesity	44	44.0	16.5	70.0	7.20	--
OB2	Obesity	41	41.0	17.8	66.7	6.21	836.2
OB3	Obesity	33	49.3	17.9	40.0	5.60	664.2
OB4	Obesity	48	36.2	15.3	100.0	2.78	788.9
OB5	Obesity	28	42.9	15.7	65.0	3.33	771.2

PA1, primary adipocyte donor 1, whose visceral adipose tissue was used for primary adipocyte isolation (Figure 5A), but not for the adipose tissue explant experiments; OW, overweight; OB, obesity.
